# Сочетание эндогенного гиперкортицизма и первичного гиперпаратиреоза: клинические и генетические особенности

**DOI:** 10.14341/probl13741

**Published:** 2026-09-08

**Authors:** Е. О. Мамедова, Е. Г. Пржиялковская, Л. Я. Рожинская, Э. А. Янар, И. С. Чугунов, А. А. Колодкина, Е. В. Васильев, А. Н. Тюльпаков, Ж. Е. Белая, Г. А. Мельниченко

**Affiliations:** Национальный медицинский исследовательский центр эндокринологии им. академика И.И. Дедова Россия; Endocrinology Research Centre Russian Federation; Медико-генетический научный центр им. академика Н.П. Бочкова; Российская детская клиническая больница ФГАОУ ВО РНИМУ им. Н.И. Пирогова Минздрава России Россия; Research Centre for Medical Genetics; Russian Children’s Clinical Hospital Russian Federation

**Keywords:** эндогенный гиперкортицизм, болезнь Иценко-Кушинга, синдром Иценко-Кушинга, АКТГ-эктопированный синдром, первичный гиперпаратиреоз, MEN1, ARMC5, endogenous hypercortisolism, Cushing’s disease, Cushing’s syndrome, ACTH-ectopic syndrome, primary hyperparathyroidism, MEN1, ARMC5

## Abstract

**ОБОСНОВАНИЕ:**

ОБОСНОВАНИЕ. Сочетание эндогенного гиперкортицизма (ЭГ) и первичного гиперпаратиреоза (ПГПТ) встречается редко, в литературе описаны единичные клинические случаи. Причины развития такого сочетания остаются малоизученными.

**ЦЕЛЬ:**

ЦЕЛЬ. Изучить клинические и генетические особенности сочетания ЭГ и ПГПТ.

**МАТЕРИАЛЫ И МЕТОДЫ:**

МАТЕРИАЛЫ И МЕТОДЫ. Проведено ретроспективное одноцентровое одномоментное наблюдательное исследование, в котором проанализированы клинические признаки пациентов с сочетанием ЭГ и ПГПТ. Всем пациентам ранее проводилось молекулярно-генетическое исследование: секвенирование по Сэнгеру гена MEN1 (n=10), высокопроизводительное параллельное секвенирование панели, включавшей гены MEN1 и CDKN1B (n=3), полноэкзомное секвенирование (n=8).

**РЕЗУЛЬТАТЫ:**

РЕЗУЛЬТАТЫ. В исследование включен 21 пациент (17 женщин и 4 мужчин): 17 – с болезнью Иценко-Кушинга (БИК) и ПГПТ, 3 пациентки с синдромом Иценко-Кушинга (СИК) и ПГПТ, и одна пациентка с АКТГ-эктопированным синдромом (АКТГ-ЭС) и ПГПТ. В группе пациентов с БИК и ПГПТ у 10 имелись мутации в MEN1 (синдром множественных эндокринных неоплазий 1 типа (МЭН-1)), у 7 не было выявлено мутаций (фенокопии МЭН-1). У пациентов с МЭН-1 дебют и БИК, и ПГПТ был в более молодом возрасте по сравнению с фенокопиями МЭН-1 (p=0,015 и p=0,0006). У 60% пациентов с МЭН-1 дебют БИК был в детском возрасте, и у всех детей БИК была первым проявлением синдрома. У 75% взрослых пациентов с МЭН-1 БИК была диагностирована после ПГПТ, тогда как у всех фенокопий МЭН-1 ПГПТ был диагностирован после или при обследовании по поводу БИК. У большинства пациентов с МЭН-1 имелись также гастро-энтеро-панкреатические нейроэндокринные опухоли (НЭО), у части – НЭО легких, у пациентов с фенокопиями МЭН-1 НЭО отсутствовали. У одной пациентки с СИК (двустороннее поражение надпочечников) и ПГПТ была выявлена мутация в гене ARMC5. У пациентки с АКТГ-ЭС и ПГПТ мутаций не выявлено.

**ЗАКЛЮЧЕНИЕ:**

ЗАКЛЮЧЕНИЕ. Сочетание ЭГ и ПГПТ чаще встречается у женщин. У детей с МЭН-1 и у фенокопий МЭН-1 первым проявлением синдрома чаще является БИК, а ПГПТ диагностируют случайно в ходе обследования, тогда как у взрослых пациентов с МЭН-1 первым проявлением чаще является ПГПТ. Причинами сочетания двух опухолевых эндокринных заболеваний могут быть мутации в гене MEN1, а в случае СИК и ПГПТ – возможно, в гене ARMC5, однако в большинстве случаев причина остается неясной. Выяснение причин сочетанного развития ЭГ и ПГПТ может расширить представления о механизмах развития эндокринных опухолей.

## Abstract (en)

**BACKGROUND:**

BACKGROUND: A combination of endogenous hypercortisolism and primary hyperparathyroidism (PHPT) occurs rarely, few clinical cases are described in the literature. The causes of the development of such combination are poorly investigated. AIM: To study clinical and genetic characteristics of a combination of endogenous hypercortisolism and PHPT.

**MATERIALS AND METHODS:**

MATERIALS AND METHODS: A retrospective, single-center, cross-sectional, observational study was performed. Clinical characteristics of patients with a combination of endogenous hypercortisolism and PHPT were analyzed. All patients had previously undergone genetic testing as follows: MEN1 Sanger sequencing (n=10), next-generation sequencing of a panel of genes including MEN1 and CDKN1B (n=3), whole-exome sequencing (n=8).

**RESULTS:**

RESULTS: 21 patients (17 females, 4 males) were included in the study: 17 with Cushing’s disease (CD) and PHPT, 3 patients with Cushing’s syndrome (CS) and PHPT, and one patient with ACTH-ectopic syndrome (ACTH-ES) and PHPT. Among patients with CD and PHPT ten had MEN1 mutations (multiple endocrine neoplasia type 1 syndrome (MEN1)), and seven did not have MEN1 mutations (MEN1 phenocopies). In patients with MEN1 the debut of both CD and PHPT occurred at younger age in comparison to MEN1 phenocopies (p=0,015 and p=0,0006). In 60% of MEN1 CD occured in infancy, and in all children CD was the first manifestation. In 75% of adult MEN1 patients CD was diagnosed after PHPT, whereas in all MEN1 phenocopies PHPT was diagnosed after or during investigation with regard to CD. The majority of MEN1 patients also had gastro-entero- pancreatic neuroendocrine tumors (NETs), and some had lung NETs, whereas patients with MEN1 phenocopies did not have NETs. One patient with CS (bilateral lesions) and PHPT had ARMC5 mutation. In a patient with ACTH-ES and PHPT no mutations were found.

**CONCLUSION:**

CONCLUSION: A combination of endogenous hypercortisolism and PHPT occurs more frequently in females. In children with MEN1 and in MEN1 phenocopies the first manifestation is more frequently CD, while PHPT is diagnosed accidentally during evaluation, whereas in adults with MEN1 the first manifestation is more frequently PHPT. MEN1 mutations can be the cause of a combination of these two endocrine tumor diseases, and, possibly, ARMC5 mutations in cases of CS and PHPT, though in the majority of cases the cause remains unknown. Identification of causes of endogenous hypercortisolism and PHPT co-occurrence can expand our understanding of the mechanisms of endocrine tumor development.

## ОБОСНОВАНИЕ

Эндогенный гиперкортицизм (ЭГ) — редкое заболевание, обусловленное длительным воздействием на организм избыточного количества кортикостероидов, в этиологической структуре которого преобладают АКТГ (адренокортикотропный гормон)-зависимые формы (болезнь Иценко-Кушинга (БИК) — 60–70%, АКТГ-эктопический синдром (АКТГ-ЭС) — 5–10%), тогда как первичное поражение надпочечников (синдром Иценко-Кушинга, СИК) составляет 20–30% [1]. В структуре СИК выделяют одностороннее (аденома надпочечника (75–90%) или адренокортикальный рак (<5%)) и двустороннее поражение надпочечников (10%) [2]. Двустороннюю гиперплазию надпочечников в зависимости от размера узлов подразделяют на микро-и макронодулярную, к последней относят первичную двустороннюю макронодулярную гиперплазию надпочечников (ПДМГН) [3]. Первичный гиперпаратиреоз (ПГПТ) — эндокринное заболевание, обусловленное формированием очага хронической гиперпродукции паратиреоидного гормона (ПТГ) (в 85% случаев — вследствие аденомы одной околощитовидной железы (ОЩЖ), в 15% случаев — аденомы/гиперплазии нескольких ОЩЖ, менее 1% случаев — карциномы ОЩЖ) [4]. К синдромам, в рамках которых могут одновременно возникать и ЭГ, и ПГПТ, относятся синдром множественных эндокринных неоплазий 1 типа (МЭН-1) (как АКТГ-зависимые, так и АКТГ-независимые формы) [5] и синдром множественных эндокринных неоплазий 4 типа (МЭН-4) (только БИК) [6]. Синдром МЭН-1, проявляющийся сочетанным развитием ПГПТ, аденом гипофиза и гастро-энтеро-панкреатических нейроэндокринных опухолей (НЭО), возникает в результате герминальных мутаций в гене-супрессоре опухолевого роста MEN1. Клинический диагноз синдрома основывается на сочетании минимум двух из трех вышеперечисленных опухолей и должен быть подтвержден результатами молекулярно-генетического исследования [7][8]. Случаи сочетания минимум двух МЭН-1-ассоциированных опухолей у одного пациента при отсутствии мутации в гене MEN1 обозначаются как фенокопии МЭН-1. У таких пациентов чаще встречается сочетание аденом гипофиза и ПГПТ, а среди аденом гипофиза преобладают соматотропиномы [9]. Синдром МЭН-4 является фенокопией МЭН-1, поскольку клинические проявления синдромов во многом схожи, но его причиной являются герминальные мутации в гене CDKN1B [6][9]. Кроме того, описано несколько случаев сочетания ПДМГН и ПГПТ у пациентов с мутациями в гене ARMC5 [10–12]. Другие причины сочетания ЭГ и ПГПТ у одного пациента в настоящее время неизвестны.

## ЦЕЛЬ ИССЛЕДОВАНИЯ

Изучение клинических и генетических особенностей пациентов с сочетанием ЭГ и ПГПТ.

## МАТЕРИАЛЫ И МЕТОДЫ

## Место и время проведения исследования

Место проведения. ГНЦ РФ ФГБУ «НМИЦ эндокринологии им. академика И.И. Дедова» Минздрава России, отделение нейроэндокринологии и остеопатий, с 2022 г. — отделение нейроэндокринологии и отделение остеопороза и остеопатий, Институт детской эндокринологии.

Время исследования. Пациенты старше 18 лет — 2010–2025 гг., дети — 1992–2025 гг.

## Изучаемые популяции (одна или несколько)

Популяция: пациенты с сочетанием лабораторно подтвержденного ЭГ и ПГПТ.

Критерии включения: лабораторно подтвержденные диагнозы ЭГ и ПГПТ у лиц любого возраста, обоего пола.

## Способ формирования выборки из изучаемой популяции (или нескольких выборок из нескольких изучаемых популяций)

Сплошной.

## Дизайн исследования

Ретроспективное одноцентровое одномоментное наблюдательное.

## Методы

Из базы данных пациентов с генетически доказанным синдромом МЭН-1, состоящей на момент 2025 г. из 90 человек, отобрано 7, у которых имелось сочетание БИК и ПГПТ. Из базы данных пациентов с фенокопиями МЭН-1, состоящей из 76 пациентов с сочетанием аденомы гипофиза и ПГПТ, отобрано 7, у которых имелось сочетание БИК и ПГПТ. Из базы данных детей с БИК, состоящей из 131 пациента, отобрано 3, у которых имелось сочетание БИК и ПГПТ. Также в исследование включены 3 пациентки с СИК и ПГПТ, и 1 пациентка с сочетанием АКТГ-ЭС и ПГПТ.

Всем пациентам ранее проводилось молекулярно-генетическое исследование, на основании чего они были отнесены к группам генетически доказанного МЭН-1 и фенокопий МЭН-1, в следующем объеме: секвенирование по Сэнгеру гена MEN1 (n=10); высокопроизводительное параллельное секвенирование (NGS) панели генов-кандидатов (Ion Torrent PGM, Thermo Fisher Scientific — Life Technologies, США), включавшей гены AIP, CASR, CDKN1A, CDKN1B, CDKN1C, CDKN2A, CDKN2C, CDKN2D, DICER1, GNAS, CDC73, MEN1, POU1F1, PRKAR1A, PRKCA, PTTG2, SDHA, SDHB, SDHC, SDHD (n=3); полноэкзомное секвенирование (NextSeq550, Illumina, США) (n=8). Методология исследований описана нами ранее [4][10]). Диагнозы ЭГ и ПГПТ устанавливались в соответствии с клиническими рекомендациями [13][14]. Оценивались возраст на момент диагноза обоих заболеваний, данные топической диагностики, результаты лечения, данные гистологического исследования, наличие осложнений обоих заболеваний, наличие других опухолей.

## Статистический анализ

Предварительный расчет размера выборки не проводился. Статистическая обработка данных проводилась с использованием пакета прикладных программ TIBCO® Statistica™ Version 13.3. Для описания количественных признаков использовались медиана, 1-й и 3-й квартили, для описания качественных признаков — доли. Для сравнения количественных признаков использовался критерий Манна-Уитни, для качественных — точный двусторонний критерий Фишера. Различия показателей считались статистически значимыми при уровне значимости меньше 0,05.

## Этическая экспертиза

Протокол исследования одобрен на заседании локального этического комитета ГНЦ РФ ФГБУ «НМИЦ эндокринологии им. академика И.И. Дедова» Минздрава России от 26.06.2024 г., протокол №12.

## РЕЗУЛЬТАТЫ

## Сочетание БИК и ПГПТ

Характеристика пациентов с сочетанием БИК и ПГПТ (n=17) представлена в таблице 1. В группе БИК и ПГПТ у 10 пациентов были выявлены мутации в гене MEN1 (генетически подтвержденный МЭН-1), у 7 — не выявлены (фенокопии МЭН-1). У 14 пациентов были микроаденомы гипофиза, у одного — макроаденома гипофиза, у двух данные о размере аденомы исходно отсутствовали. Среди пациентов с МЭН-1 — 6 женского пола, 4 — мужского. У 6 (60%) дебют БИК был в детском возрасте (до 18 лет). Средний возраст на момент диагноза БИК составил 15,5 года [ 13; 33], на момент диагноза ПГПТ — 28 лет [ 18; 33]. У 4 пациентов БИК была диагностирована раньше ПГПТ, у 3 ПГПТ был диагностирован одновременно при обследовании по поводу БИК, у 3 пациентов ПГПТ был диагностирован раньше БИК. Среди пациентов с фенокопиями МЭН-1 (n=7, все — женщины) средний возраст на момент диагноза БИК составил 50,5 года [ 43; 57], на момент диагноза ПГПТ 56 лет [ 54; 59]. У всех пациенток с фенокопиями МЭН-1 БИК была либо первым проявлением синдрома, а ПГПТ был диагностирован позже, либо ПГПТ был выявлен случайно при обследовании по поводу БИК. Возраст диагноза БИК у пациентов с МЭН-1 и фенокопиями МЭН-1 статистически значимо отличался (p=0,015), также как и возраст диагноза ПГПТ (p=0,0006). У двух из четырех пациентов с МЭН-1 (пациенты 1.3 и 1.4) диагноз ЭГ был установлен лабораторно, на основании неоднократно зафиксированных повышений кортизола в слюне вечером, кортизола в крови вечером, кортизола в суточной моче и отсутствия подавления кортизола на фоне ночного подавляющего теста с 1 мг дексаметазона. При этом явных клинических проявлений ЭГ у пациентов не было, на основании чего от проведения трансназальной транссфеноидальной аденомэктомии решили воздержаться. Кроме того, у одного пациента (1.4) имелось значимое повышение уровня инсулиноподобного фактора роста 1, без клинических проявлений акромегалии. У одной пациентки (1.2) исходно была диагностирована пролактинома, а БИК манифестировала гораздо позже (клинический случай подробно описан ранее [15]). У 8 (80%) пациентов с МЭН-1 имелось поражение более одной ОЩЖ, тогда как у всех пациентов с фенокопиями МЭН-1 имелись солитарные образования ОЩЖ (p=0,0023). Также у 9 (90%) пациентов с МЭН-1 на момент включения в исследование имелись данные о наличии гастро-энтеро-панкреатических НЭО, у 4 пациентов — НЭО легких, тогда как у пациентов с фенокопиями МЭН-1 не было ни одного случая НЭО (p=0,0004). У одной пациентки с фенокопией МЭН-1 (2.1) был выявлен папиллярный рак щитовидной железы, у нее же выявлен вариант с неопределенной клинической значимостью в гене CDKN1A (табл. 1). У всех пациентов имелись осложнения, при этом следует отметить, что остеопороз и мочекаменная болезнь могут быть одновременно осложнениями и ЭГ, и ПГПТ.

**Table:** Таблица 1. Характеристика пациентов с БИК и ПГПТ Примечания: * — лабораторно, ** — исходно у пациентки в 20 лет диагностирована пролактинома, проводилась ТА; данные указаны на момент диагностики ЭГ (клинический случай подробно описан в статье [15]), *** — динамическое наблюдение по поводу БИК ввиду отсутствия явных клинических проявлений ЭГ, Ж — женщина, М — мужчина, микро — микроаденома гипофиза (<1 см), макро — макроаденома гипофиза (≥1 см), NGS — высокопроизводительное параллельное секвенирование, АГ — артериальная гипертензия, АКТГ — адренокортикотропный гормон, БИК — болезнь Иценко-Кушинга, ВНЗ — вариант с неопределенной клинической значимостью, ИБС — ишемическая болезнь сердца, ИМ — инфаркт миокарда, ЛТ — лучевая терапия, МКБ — мочекаменная болезнь, МСКТ — мультиспиральная компьютерная томография, Н/д — нет данных, НЭО — нейроэндокринная опухоль, ОНМК — острое нарушение мозгового кровообращения, ОП — остеопороз, ОЩЖ — околощитовидные железы, ПГПТ — первичный гиперпаратиреоз, ПРЛ — пролактин, ПТЭ — паратиреоидэктомия, СД — сахарный диабет, СТГ — соматотропный гормон, ТА — трансназальная аденомэктомия, УЗИ — ультразвуковое исследование, ХТ — химиотерапия.

Синдром МЭН-1
Пациент	Пол	БИК	ПГПТ	Осложнения	Другие опухоли (лечение)	Генетическое исследование, результат
Возраст на момент диагноза, лет	Размер аденомы гипофиза (микро/макро)	Тип секреции*	Лечение	Гистология	Ремиссия (да/нет)	Возраст на момент диагноза, лет	Топическая диагностика (УЗИ и/или сцинтиграфия с технетрилом)	Лечение	Гистология	Ремиссия (да/нет)
1.1	Ж	33	Микро	АКТГ	ТА, ЛТ	Н/д	Нет	33	Образования левых верхней и нижней, правой и правой верхней ОЩЖ	Удаление трех аденом ОЩЖ	Аденомы	Нет (эктопированные в средостение три образования ОЩЖ)	СД, АГ, ОП	•НЭО поджелудочной железы, гормонально-неактивные (аналоги соматостатина)•НЭО S8 правого легкого с mts в печень (ХТ) (ex. let.)	Секвенирование по Сэнгеру гена MEN1(NM_130799.2)c.332delG p.Gly111Valfs*8Патогенный
1.2**	Ж	42	Микро	ПРЛ/АКТГ	ТА	Аденома гипофиза	Да	39	Образования четырех ОЩЖ	Тотальная ПТЭ	Н/д	Да	СД, МКБ, ОП	• Гастриномы (хирургическое лечение)• НЭО поджелудочной железы, гормонально-неактивные (динамическое наблюдение)	Секвенирование по Сэнгеру гена MEN1(NM_130799.2)с.231С>А р.Tyr77*Патогенный
1.3	Ж	33	Микро	АКТГ	Нет***	-	Нет	28	Образования правой и левой верхних и правой нижней ОЩЖ	Двукратно ПТЭ (удалены правая и левая верхние и правая нижняя ОЩЖ)	Н/д	Да	МКБ	• НЭО поджелудочной железы, гормонально-неактивные (аналоги соматостатина)• НЭО S1+2 левого легкого (хирургическое лечение)• Липома левой лопаточной области, нижней трети плеча	Секвенирование по Сэнгеру гена MEN1(NM_130799.2)с.1561dupC p.Arg521Profs*15Патогенный
1.4	М	39	Микро	ПРЛ/СТГ/АКТГ	Каберголин по поводу пролактиномы***	-	Нет	33	Образования правых верхней, нижней и левой верхней ОЩЖ	Удаление трех аденом ОЩЖ	Аденомы	Да	МКБ	• НЭО поджелудочной железы, гормонально-неактивные (аналоги соматостатина)• НЭО S6 правого легкого (хирургическое лечение)	Секвенирование по Сэнгеру гена MEN1(NM 000244.3)c.202_206dupGCCCCp.Asp70Profs*51Патогенный
1.5	Ж	11	Микро	АКТГ	ЛТ	Н/д	Да	18	Образования левой и правой верхней, правой нижней ОЩЖ	Тотальная паратиреоидэктомия с аутотрансплантацией фрагмента правой нижней ОЩЖ + удаление аденомы правой нижней ОЩЖ через 9 лет	Аденомы	Нет (эктопированные в средостение три образования ОЩЖ)паратироматоз	Вторичный гипотиреоз	• НЭО поджелудочной железы, гормонально-неактивные (дистальная резекция поджелудочной железы)	Секвенирование по Сэнгеру гена MEN1(NM_130799.2)1365+1-11delGTGAGGGACAG Патогенный
1.6	М	15	Микро	АКТГ	ТА	Аденома гипофиза	Да	15	Образование правой нижней ОЩЖ	Наблюдение	-	-	Несахарный диабет	• НЭО поджелудочной железы, с гиперсекрецией вазоактивного интестинального пептида, панкреатического полипептида, глюкагона (радикальная корпокаудальная резекция поджелудочной железы)• Объемное образование S6 правого легкого	Секвенирование по Сэнгеру гена MEN1(NM_130799.2)с.1546delC: p.Arg516Glyfs*43Патогенный
1.7	Ж	13	Микро	АКТГ	ТА+ повторная ТА	Аденома гипофиза	Да	13	Образование левой верхней ОЩЖ	Наблюдение	-	-	Гипопитуитаризм: СТГ-дефицит, вторичный гипокортицизм, вторичный гипотиреоз, парциальный несахарный диабет. Гипогонадизм. Нормогонадотропная аменорея	-	Секвенирование по Сэнгеру гена MEN1(NM_130799.2)c.1646del (p.Pro549Glnfs*10)Патогенный
1.8	М	15	Микро	АКТГ	Двустороння адреналэктомия по жизненным показаниям, протонотерапия	Н/д	Да	28	Образования левой верхней и левой нижней ОЩЖ	Двукратная ПТЭ	Аденомы	Да	ОП	Н/д	Секвенирование по Сэнгеру гена MEN1(NM_130799.2)c.628_631delACAG p.S210fs*222Патогенный
1.9	М	16	Микро	АКТГ	Двукратная протонотерапия	Н/д	Да	20	Образования четырех ОЩЖ	Субтотальная ПТЭ	Н/д	Да	МКБ, ОП	• НЭО поджелудочной железы (дистальная резекция поджелудочной железы)	Секвенирование по Сэнгеру гена MEN1(NM_130799.2)c.548G>A p.Trp183*Патогенный
1.10	Ж	13	Н/д	АКТГ	Транскраниальная аденомэктомия, ТА, ЛТ, двусторонняя адреналэктомия	Аденома гипофиза	Да	28	Образования правой верхней, правой нижней, левой нижней ОЩЖ	Двукратная ПТЭ	2 – н/д, 1 – аденома	Да	СД, АГ, ОП	•НЭО поджелудочной железы (аналоги соматостатина)	Полноэкзомное секвенирование(NM_001370259.2)c.248delp.Leu83Argfs*36Вероятно патогенный
Фенокопии МЭН-1
2.1	Ж	56	Микро	АКТГ	ТА	Аденома гипофиза из базофильных клеток	Да	56	Образование левой верхней ОЩЖ	ПТЭ	Аденома из главных клеток	Да	СД, АГ	• Папиллярный рак щитовидной железы T1aN0M0 (хирургическое лечение)	NGS панели-генов-кандидатовГерминальный гетерозиготный вариант в гене CDKN1A(NM 001291549)c.452G>Ap.Cys151TyrВНЗ
2.2	Ж	56	Микро	АКТГ	ТА	Аденома гипофиза из базофильных клеток	Да	56	Образование правой нижней ОЩЖ	ПТЭ	Аденома из главных клеток	Да	СД, АГ, ОП	Нет	NGS панели-генов-кандидатовНе обнаружено
2.3	Ж	39	Микро	АКТГ	ТА	Аденома гипофиза из круковских клеток	Да	40	Образование правой нижней ОЩЖ	ПТЭ	Аденома из главных клеток	Да	СД, АГ, ОП, гипокалиемия	Нет	Полноэкзомное секвенированиеНе обнаружено
2.4	Ж	59	Макро	АКТГ	ТА	Аденома гипофиза из базофильных клеток	Да	59	Образование левой верхней ОЩЖ	Не оперирована	-	Нет	СД, АГ, ОП	Нет	Полноэкзомное секвенированиеНе обнаружено
2.5	Ж	41	Микро	АКТГ	ЛТ	Н/д	Да	67	Образование левой верхней ОЩЖ	Не оперирована	-	Нет	СД, АГ, ИБС, ИМ, МКБ	Нет	Полноэкзомное секвенированиеНе обнаружено
2.6	Ж	45	Н/д	АКТГ	2 ТА, ЛТ	Н/д	Да	57	Образование правой верхней ОЩЖ	ПТЭ	Гиперплазия ОЩЖ	Да	АГ	Нет	NGS панели-генов-кандидатовНе обнаружено
2.7	Ж	45	Микро	АКТГ	2 ТА, ЛТ	Опухоль гипофиза не обнаружена	Нет	54	Образование правой нижней ОЩЖ	Не оперирована	-	Нет	СД, АГ, ОП, ОНМК	Нет	Секвенирование по Сэнгеру гена MEN1Не обнаружено

## Сочетание СИК и ПГПТ

Характеристика трех пациенток с сочетанием СИК и ПГПТ представлена в таблице 2. У пациентки 3.1 ПГПТ был выявлен при случайном обследовании, и был первым диагностированным проявлением синдрома. Объемное образование надпочечника было выявлено также при случайном обследовании, и при оценке гормонального статуса был подтвержден диагноз АКТГ-независимого ЭГ. У нее был выявлен вариант с неопределенной клинической значимостью в гене TP53 (табл. 2). У пациентки 3.2 ПГПТ был выявлен случайно в ходе обследования по поводу ЭГ. У обеих пациенток не было подозрительного в отношении МЭН-1 семейного анамнеза и не было других опухолей. У пациентки 3.3 с сочетанием ПДМГН и ПГПТ выявлена мутация в гене ARMC5, клинический случай описан нами ранее [10]. За период наблюдения после публикации статьи пациентка была прооперирована по поводу ПГПТ в возрасте 44 лет (по гистологии — аденома), с достижением ремиссии ПГПТ.

**Table:** Таблица 2. Характеристика пациентов с СИК и ПГПТ Примечания: ВНЗ — вариант с неопределенной клинической значимостью; Ж — женщина; М — мужчина; МКБ — мочекаменная болезнь; МСКТ — мультиспиральная компьютерная томография; НП — надпочечник; ОЩЖ — околощитовидные железы; СИК — синдром Иценко-Кушинга; ПГПТ — первичный гиперпаратиреоз; ПДМГН — первичная двусторонняя макронодулярная гиперплазия надпочечников; ПТЭ — паратиреоидэктомия.

Пациент	Пол	СИК	ПГПТ	Осложнения	Другие опухоли (лечение)	Генетическое исследование, результат
Возраст на момент диагноза, лет	Топическая диагностика (МСКТ)	Лечение	Гистология	Ремиссия (да/нет)	Возраст на момент диагноза, лет	Топическая диагностика (УЗИ и/или сцинтиграфия с технетрилом)	Лечение	Гистология	Ремиссия (да/нет)
3.1	Ж	33	Образование левого НП 24х26х26 мм, неоднородной структуры, плотностью в нативную фазу от -21 до 48 HU	Левостороння адреналэктомия	Светлолеточная аденома	Да	41	Объемное образование правой нижней ОЩЖ	ПТЭ	Аденома	Нет (сохраняется образование в той же локализаци)	Нет	Нет	Полноэкзомное секвенированиеГерминальный гетерозиготный вариант в гене TP53(NM_000546.6)c.472C>T (p.Arg158Cys)ВНЗВариант встречается в популяционной базе данных gnomAD v2.1.1 в гетерозиготном состоянии с частотой 0.0007959%.Вариант расположен в неконсервативной позиции.
3.2	Ж	52	Образование левого НП 41х32х46 мм. Плотность по фазам: нативная-артериальная-венозная-отсроченная: 36-68-88-61 HU Узелковая гиперплазия правого НП	Левостороння адреналэктомия	Не предоставлена	Да	53	Объемное образование левой верхней ОЩЖ	ПТЭ	Аденома	Да	Гипокалиемия, МКБ (микролиты)	Нет	Полноэкзомное секвенированиеНе обнаружено
3.3	Ж	37	Правый НП резко увеличен в размерах до 8,7х3,2х3,3 см, за счет множественных кистовидных образований до 3,0 см, плотностью в пределах 25-28 HUЛевый НП размерами 6.4*2,3*2,7 см неправильной формы за счет аналогичных кистозных изменений диаметром 1.8-2,5 см, плотностью 17-26 HU	Двусторонняя адреналэктомия	ПДМГН	Да	37	Объемное образование правой верхней ОЩЖ	ПТЭ	Аденома	Да	МКБ (микролиты)	Нет	Полноэкзомное секвенированиеГерминальный гетерозиготный ва-риант в экзоне 6 гена ARMC5(NM_001105247)c.2692C>Tp.Arg898TrpПатогенный

## Пациентка с сочетанием АКТГ-ЭС и ПГПТ

У пациентки в возрасте 62 лет был диагностирован ЭГ тяжелого течения (кортизол в крови вечером 1468 нмоль/л (64–327), кортизол слюны вечером 162,2 нмоль/л (0,5–9,65), кортизол в суточной моче 2365,5 нмоль/сут (100–379), АКТГ утром 98,89 пг/мл (7,2–63,3), АКТГ вечером 86,06 пг/мл (2–25,5)). По МСКТ органов грудной клетки в корне левого легкого выявлено округлое образование размерами 31х29х35 мм, плотностью по фазам сканирования 40-40-165-60 HU (нативная-артериальная-венозная-отсроченная), с четкими ровными контурами, выраженно деформирующее верхнедолевой и субсегментарные бронхи верхней доли левого легкого. Также на МСКТ параэзофагеально справа выявлено образование овальной формы размерами 21х16х23 мм, с четкими ровными контурами, плотностью по фазам сканирования 40-40-170-60 HU. Паратрахеально справа отмечена структура овальной формы с четкими контурами размерами 7,5х3,5 мм, плотностью по фазам сканирования 26-65-225-45 HU. Из осложнений выявлены сахарный диабет, артериальная гипертензия, мочекаменная болезнь и остеопороз. Пациентке проведена верхняя лобэктомия слева с медиастинальной лимфодиссекцией, по гистологии — НЭО сT2aN0M0. После операции лабораторно достигнута ремиссия ЭГ, однако сохранялся увеличенный паратрахеальный лимфоузел справа, пациентке назначены аналоги соматостатина длительного действия. Одновременно с ЭГ диагностирован ПГПТ (кальций общий 3,23 ммоль/л (2,15–2,55), паратгормон 525 пг/мл (15–65)). После лечения ЭГ пациентке проведено удаление образования правой верхней ОЩЖ, при гистологическом исследовании — онкоцитарная аденома ОЩЖ. После операции достигнута ремиссия ПГПТ. Пациентке проведено полноэкзомное секвенирование, по результатам которого патогенных вариантов не обнаружено.

## ОБСУЖДЕНИЕ

Сочетание ЭГ и ПГПТ встречается достаточно редко. Первые предположения о возможном сочетании этих двух состояний были выдвинуты в начале — первой половине XX века (описано около 8 случаев) [цитируется по 16], однако подробное клинико-лабораторное описание двух пациентов было сделано Raker и соавт. в 1962 г. [16]. Они описали двух женщин 43 и 23 лет, соответственно с сочетанием СИК вследствие двусторонней нодулярной гиперплазии надпочечников и ПГПТ вследствие 5-см аденомы ОЩЖ, расположенной позади дуги аорты, и сочетанием СИК вследствие гиперплазии левого надпочечника и ПГПТ вследствие аденомы левой ОЩЖ большого размера [16]. В доступной литературе на английском языке описаны единичные случаи сочетания СИК и ПГПТ [17–19]. Сочетание БИК и ПГПТ исторически было описано в рамках синдрома МЭН-1 [20][21].

В исследовании Simonds и соавт. были проанализированы характеристики 19 пациентов с МЭН-1 и ЭГ (8 мужчин, 11 женщин); пациенты были набраны ретроспективно за около 40-летний период и отобраны среди 120 пациентов с МЭН-1 [22]. Из них этиология заболевания была установлена у 14 — 11 БИК и 3 СИК. Средний возраст на момент установки диагноза ЭГ составил 36±15,9 года. Среди пациентов с БИК было три ребенка с возрастом на момент дебюта заболевания 10, 14 и 15 лет. У 3 из 11 пациентов с БИК (27%) при хирургическом вмешательстве были обнаружены дополнительно гормонально-неактивные микроаденомы гипофиза, что встречается в 10 раз реже при спорадической БИК. У 2 из 3 пациентов с СИК был адренокортикальный рак [22]. Позднее Simonds в своем обзоре суммировал основные характеристики ЭГ в рамках МЭН-1 [5]. Примечательно, что БИК в рамках МЭН-1 может быть первым проявлением синдрома у детей и подростков: в обзоре представлены характеристики 10 таких пациентов, описанных в литературе [5]. В российской работе Янар Э.А. среди 35 детей и подростков с БИК у 4 (11%) были выявлены мутации в гене MEN1 [23]. У 40% пациентов с МЭН-1 имеются образования надпочечников, но большинство из них являются гормонально-неактивными [7]. В работе Wang и соавт. было показано, что среди пациентов с МЭН-1, имевших образования надпочечников, у 25% был ЭГ [24]. АКТГ-ЭС у пациентов с МЭН-1 встречается крайне редко и описан в зарубежной литературе только вследствие карциноидов тимуса, имеющих в большинстве случаев агрессивное течение, а к факторам риска относят мужской пол и курение [25–28]. В российской литературе описан случай клинического МЭН-1 (без генетической верификации) с АКТГ-ЭС вследствие АКТГ-продуцирующей феохромоцитомы [29].

Синдром МЭН-4 часто обозначают как фенокопию синдрома МЭН-1, поскольку клиническая картина обоих синдромов схожа [30], однако МЭН-4 встречается гораздо реже МЭН-1 [6]. Среди описанных в литературе 48 пациентов с МЭН-4 с мутациями в гене CDKN1B у 7 была диагностирована БИК, при этом у двух БИК сочеталась с ПГПТ (возраст 37 и 46 лет момент диагноза БИК и 36 и 47 лет на момент диагноза ПГПТ соответственно), а остальные 5 пациентов были дети с БИК [6]. Таким образом, МЭН-4 может быть еще одной причиной БИК в детском возрасте [31].

В нашем исследовании распределение АКТГ-зависимых и независимых форм ЭГ в целом соответствовало популяционным. Возраст дебюта БИК был ниже у пациентов с МЭН-1 по сравнению с фенокопиями МЭН-1. Учитывая данные Янар Э.А., можно сделать вывод, что дебют БИК в детском и подростковом возрасте требует исключения синдрома МЭН-1, а учитывая данные литературы — и МЭН-4. Примечательно, что в нашей выборке у двух пациентов с МЭН-1 ЭГ не проявлял себя клинически, а в одном случае имела место трансформация пролактиномы в кортикотропиному. В нашем исследовании не было выявлено ни одного пациента с мутацией в гене CDKN1B, что подчеркивает редкость МЭН-4. Также мы не выявили ни одного пациента с МЭН-1 и СИК. У единственной пациентки с АКТГ-ЭС и ПГПТ также не было выявлено мутаций в MEN1, однако, как отмечено выше, в литературе АКТГ-ЭС в рамках МЭН-1 встречался только вследствие карциноидов тимуса и, возможно, феохромоцитомы. Примечательно, что у единственной пациентки с фенокопией МЭН-1 и вариантом с неопределенной клинической значимостью в гене CDKN1A также был выявлен папиллярный рак щитовидной железы, однако является ли этот вариант причиной развития всех трех опухолей, неясно. Мутации в гене TP53 приводят к развитию синдрома Ли-Фраумени, характеризующимся развитием адренокортикального рака, рака молочной железы, колоректального рака, сарком и др., в том числе в молодом возрасте [32], однако у пациентки 3.1, у которой был выявлен вариант с неопределенной клинической значимостью в гене TP53, отсутствуют другие опухоли, характерные для этого синдрома.

В 2019 г. нами была описана пациентка с сочетанием первичной двусторонней макронодулярной гиперплазии надпочечников и ПГПТ, и мутацией в гене ARMC5 [10]. В статье Gagliardi и соавт. у одного пациента с ПДМГН (семейная форма) и мутацией в гене ARMC5 был выявлен ПГПТ, однако соматических мутаций в гене ARMC5 в ткани аденомы ОЩЖ выявлено не было [33]. В литературе описан еще один случай сочетания ПДМГН вследствие мутации в ARMC5 и атипично расположенной около дуги аорты аденомы ОЩЖ, однако хирургическое лечение ПГПТ не проводилось [34]. Нашими коллегами также описан случай сочетания ПДМГН и ПГПТ с множественным поражением ОЩЖ и рецидивом ПГПТ после хирургического лечения, однако при полноэкзомном секвенировании патогенных вариантов не выявлено [35]. С учетом, что у пациентов с мутациями в ARMC5 описано также развитие менингиом и акромегалии [33], не исключено, что эти мутации предрасполагают к развитию множественных опухолей. Приводят ли мутации в ARMC5 к развитию ПГПТ, в настоящее время неизвестно.

Одной из предполагаемых причин развития множественных опухолей без выявленных мутаций может быть подавление экспрессии или нарушение регуляции экспрессии генов, ассоциированных с синдромом МЭН-1, в частности на уровне посттрансляционной регуляции вследствие изменения экспрессии микроРНК [36][37]. Так, при сравнении профиля микроРНК периферической крови пациентов с генетически подтвержденным МЭН-1 и его фенокопиями было выявлено четкое разделение этих заболеваний по спектру микроРНК, исследованных методом NGS, которое было верифицировано и менее чувствительной ПЦР в реальном времени, при этом были выявлены различия по микроРНК, участвующих в регуляции экспрессии CDKN1B [38]. В других исследованиях были выявлены микроРНК, влияющие на экспрессию MEN1 [39][40].

C 1980-х гг. изучалась возможная взаимосвязь между АКТГ/кортизолом и ПТГ/фосфорно-кальциевым обменом. Так, в работе Kohri и соавт. вводили 1 мг АКТГ 6 пациентам с ПГПТ, 6 пациентам с мочекаменной болезнью и 5 здоровым контролям. Через 2 часа после инъекции уровень кальция в крови статистически значимо стал выше у пациентов с ПГПТ, но не в двух других группах. Уровень ПТГ после инъекции вырос во всех трех группах. При добавлении АКТГ к культуре клеток из аденомы ОЩЖ концентрация кальция в этой культуре возрастала. Авторы предположили, что АКТГ может влиять на клетки ОЩЖ [41]. Abou-Samra и соавт. показали, что ПТГ может стимулировать выработку АКТГ на клеточной линии кортикотрофов морской свинки AtT [42]. Nakayama и соавт. продемонстрировали, что паратгормон-подобный пептид стимулирует выработку АКТГ гипофизом кур [43].

Известно, что среди фенокопий МЭН-1 преобладают сочетания соматотропином и ПГПТ [8][9]. В работе Nachtigal и соавт. было продемонстрировано, что среди пациентов с акромегалией клиническое сочетание соматотропиномы и ПГПТ без мутации в гене MEN1 встречается в 6,1% случаев (22 из 362), и у этих пациентов повышен риск других новообразований по сравнению с изолированной акромегалией [44]. Авторы предполагают, что сочетание акромегалии и ПГПТ является отдельным состоянием, которое нельзя объяснить избыточным воздействием гормона роста на ОЩЖ, и причины развития которого требуют дальнейшего изучения [44]. Является ли сочетание ЭГ и ПГПТ отдельной нозологической единицей и оба заболевания развиваются вследствие общих механизмов, или это сочетание является случайным, а также в какой последовательности появляются оба заболевания, также требует изучения. Примечательно, что в большинстве случаев ЭГ был диагностирован ранее ПГПТ. Возможно, это связано с тем, что ЭГ ярко проявляет себя клинически, а остеопороз и мочекаменная болезнь могут быть клиническими проявлениями как ЭГ, так и ПГПТ, и только определение уровня кальция у пациентов с ЭГ позволяет диагностировать ПГПТ.

## ЗАКЛЮЧЕНИЕ

Сочетание ЭГ и ПГПТ у одного пациента может объясняться мутациями в генах MEN1 (БИК и СИК) и CDKN1B (только БИК, по данным литературы). Могут ли мутации в гене ARMC5 у пациентов с ПДМГН приводить к развитию ПГПТ, требует изучения. Всем пациентам с ЭГ рекомендуется хотя бы однократно определять уровень кальция в крови c целью не пропустить ПГПТ. Является ли большинство случаев сочетания БИК и ПГПТ, СИК (одностороннее поражение) и ПГПТ, и АКТГ-ЭС и ПГПТ случайным, или же в их основе лежат общие механизмы, почему это сочетание чаще затрагивает женщин, и почему чаще манифестирует с ЭГ, также требует дальнейшего изучения.

## ДОПОЛНИТЕЛЬНАЯ ИНФОРМАЦИЯ

Источники финансирования. Исследование выполнено за счет средств гранта РНФ 24-15-00283

Конфликт интересов. Авторы декларируют отсутствие явных и потенциальных конфликтов интересов, связанных с содержанием настоящей статьи.

Участие авторов. Все авторы одобрили финальную версию статьи перед публикацией, выразили согласие нести ответственность за все аспекты работы, подразумевающую надлежащее изучение и решение вопросов, связанных с точностью или добросовестностью любой части работы.
